# 20例戈谢病遗传学特点和临床分析

**DOI:** 10.3760/cma.j.cn121090-20230506-00179

**Published:** 2024-01

**Authors:** 恬波 张, 晓玲 文, 夏林 张, 俊荣 闫, 国平 郝, 林花 杨, 睿娟 张

**Affiliations:** 1 山西医科大学第三医院（山西白求恩医院、山西医学科学院、同济山西医院），太原 030032 Third Hospital of Shanxi Medical University, Shanxi Bethune Hospital, Shanxi Academy of Medical Sciences, Tongji Shanxi Hospital, Taiyuan 030032, China; 2 宜宾市第一人民医院血液科，宜宾 644600 Department of Hematology, the First People's Hospital of Yibin, Yibin 644000, China; 3 华中科技大学同济医学院附属同济医院，武汉 430030 Tongji Hospital, Tongji Medical College, Huazhong University of Science and Technology, Wuhan 430030, China; 4 山西省儿童医院血液科，太原 030013 Department of Hematology, Shanxi Provincial Children's Hospital, Taiyuan 030013, China; 5 山西医科大学第二医院血液科，太原 030001 Department of Hematology, the Second Hospital of Shanxi Medical University, Taiyuan 030001, China

## Abstract

戈谢病（Gaucher disease，GD）是一种常染色体隐性溶酶体贮积症，具有高度异质性。本研究通过回顾性分析山西白求恩医院20例GD患者的临床表现、实验室检查、酶学及基因结果，进一步了解GD患者临床表型与基因型相关性。20例GD患者中，Ⅰ型GD16例，中位确诊年龄24岁；Ⅲ型GD 4例，中位确诊年龄19岁。所有患者均有脾大和血小板减少；16例患者有骨骼影像学改变，5例合并骨痛症状。20例患者共检出15种基因突变，多为错义突变，以L483P（35.7％）突变多见，其次为V414L、L303I、F252I，突变位点多见于7号外显子。其中，S310G突变为本课题组首次报道，K196R突变为中国人群首次报道，发现N227S突变可能与神经病变相关。GD患者临床表型与基因型之间仍存在不确定性。

## Abstract (en)

Gaucher Disease (GD) is an autosomal recessive lysosomal storage disorder characterized by high heterogeneity. This study aimed to further understand the correlation between clinical phenotypes and genotypes in GD patients through a retrospective analysis of 20 cases in Shanxi Bethune Hospital, including their clinical manifestations, laboratory tests, enzyme studies, and genetic results. Among the 20 GD patients, 16 were classified as Type Ⅰ GD with a median age of diagnosis of 24 years, and 4 were classified as Type Ⅲ GD with a median age of diagnosis of 19 years. All patients exhibited splenomegaly and thrombocytopenia, with 16 patients showing skeletal imaging changes, and 5 of them presenting with bone pain symptoms. Genetic analysis revealed 15 distinct mutations, predominantly missense mutations, with L483P being the most prevalent (35.7%), followed by V414L, L303I, and F252I. Mutation sites were predominantly located in exon 7. Noteworthy findings included the first report of the S310G mutation by our research group and the first occurrence of the K196R mutation in the Chinese population. Additionally, the N227S mutation was implicated in a potential association with neuropathy. Despite advancements, Uncertainties still exist in the correlation between clinical phenotypes and genotypes in GD patients.

戈谢病（Gaucher disease，GD）是一种常染色体隐性罕见遗传性疾病，中国人群发病率为1/50万～1/20万，根据疾病是否累及神经系统及进展速度，GD分为非神经病变型（Ⅰ型）、急性神经病变型（Ⅱ型）、慢性或亚急性神经病变型（Ⅲ型）3种类型[1]。GD的发病机制是葡萄糖脑苷脂酶（glucocerebrosidase，GBA）基因突变导致GBA活性降低或缺乏，造成葡萄糖神经酰胺无法水解，沉积在单核巨噬细胞中。最常累及肝、脾、骨及脑，临床表现为肝脾肿大、贫血、血小板减少、骨痛、骨危象和（或）神经系统受累，病情呈进行性加重，具有一定的致残性[2]。由于GD临床表现具有复杂多样性，早期识别及鉴别诊断较为困难，漏诊、误诊率高；此外，GD致病基因突变类型与临床表型关系尚未明确。我国目前尚无GD大样本流行病学数据。本研究回顾性分析了山西地区20例GD患者临床资料，并探讨GD患者的临床表型与遗传学特点。

## 病例与方法

1. 病例：选取2021年3月至2023年3月间在山西白求恩医院诊治的20例GD患者临床资料。所有患者GD诊断均符合《中国成人戈谢病诊治专家共识（2020年）》[1]。本研究通过山西白求恩医院伦理委员会批准（批件号：SBQLL-2022-012）。

2. 实验室诊断：基于干血片法（dried blood spots，DBS）检测GBA活性（参考范围1.3～22.2 µmol·L^−1^·h^−1^）和葡糖鞘氨醇（Lyso-GL1）水平（参考范围<17.4 ng/ml）。

3. 基因检测：对20例患者的GBA基因进行二代测序（NGS），分析基因突变。提取DNA用于文库建立，采用外显子捕获法检测GBA基因外显子及其侧翼序列。测序结果用Chromos Lite2.1.1软件分析，所得序列与HGMDpro数据库中的GBA基因序列进行比对。变异位点按照人类基因组变异协会所推荐的命名法则，根据美国医学遗传学与基因组学会（ACMG）变异指南进行致病性分析。

4. 统计学处理：计数资料采用例数（％）描述，计量资料采用*M*（范围）描述。

## 结果

1. 临床特征：20例患者中，男8例，女12例（例19、20为姐妹），中位确诊年龄24（1～64）岁。Ⅰ型GD 16例，中位年龄30岁；Ⅲ型GD 4例（例3、8、9、17），中位年龄19岁。临床表现：脾大（20例，100％）、血小板减少（20例，100％）、贫血（13例，65％）、骨骼受累（16例，80％）（表1）。血常规：WBC 4.7（1.7～10.1）×10^9^/L、HGB 102（39～169）g/L、PLT 42（15～80）×10^9^/L。骨髓穿刺检查15例，12例可见戈谢细胞。腹部彩超结果显示16例为巨脾，15例合并肝大。影像学提示骨骼改变16例，骨密度减低5例，股骨下端膨大、烧瓶样改变6例，股骨及髂骨异常信号或破坏11例，脊椎侧凸2例。有骨痛症状5例，骨折1例，髋关节坏死1例。神经系统受累表现为癫痫发作3例、听力丧失1例。GD合并宫颈癌1例，帕金森病（PD）1例。16例接受酶替代治疗。患者确诊时间：4例>5年，5例2～4年，11例<1年，平均确诊时间5.6年。

**表1:** 20例戈谢病患者疾病分型和临床特征

例号	年龄（岁）	性别	体征		血液系统				骨骼系统					神经系统	疾病分型
			脾大	肝大	WBC（×10^9^/L）	HGB（g/L）	PLT（×10^9^/L）	出血	骨痛	骨折	骨密度减低	烧瓶样改变	异常信号/破坏		
1	28	男	轻度	是	5.3	109	43	否	否	否	否	否	是	-	Ⅰ型
2	55	女	巨脾	否	1.7	102	35	是	否	否	否	否	是	-	Ⅰ型
3	29	男	巨脾	是	8.6	119	20	是	是	是	是	否	是	癫痫	Ⅲ型
4	46	女	巨脾	是	3.6	102	22	否	是	否	是	否	是	-	Ⅰ型
5	35	女	巨脾	否	6.5	140	15	是	是	否	是	否	是	-	Ⅰ型
6	29	男	巨脾	是	4.7	169	41	否	否	否	是	是	是	-	Ⅰ型
7	24	女	巨脾	否	6.4	118	40	是	否	否	否	否	否	-	Ⅰ型
8	16	男	巨脾	是	3.7	111	77	是	否	否	是	是	否	癫痫	Ⅲ型
9	21	女	巨脾	是	3.1	80	30	是	否	否	否	否	是	听力丧失	Ⅲ型
10	51	女	巨脾	是	6.3	39	42	是	否	否	否	否	否	-	Ⅰ型
11	32	女	巨脾	否	5.3	110	52	否	否	否	否	是	否	-	Ⅰ型
12	64	男	巨脾	是	8.4	154	60	是	是	是	否	否	是	-	Ⅰ型
13	17	男	轻度	是	4.5	132	44	否	否	否	否	是	否	-	Ⅰ型
14	42	女	巨脾	否	10.1	82	42	是	否	否	否	否	否	-	Ⅰ型
15	1	男	轻度	是	3.6	96	45	是	否	否	否	是	否	-	Ⅰ型
16	2	女	巨脾	是	4.7	60	52	是	否	否	否	是	否	-	Ⅰ型
17	15	男	轻度	是	3.6	52	80	是	是	否	否	否	是	癫痫	Ⅲ型
18	4	女	巨脾	是	4.6	98	68	是	否	否	否	否	否	-	Ⅰ型
19	4	女	巨脾	是	3.5	96	34	是	否	否	否	否	是	-	Ⅰ型
20	2	女	巨脾	是	7.1	98	59	否	否	否	否	否	是	-	Ⅰ型

注 -为无神经系统受累

2. 酶学及Lyso-GL1检测：Ⅰ型患者GBA活性0.44（0.23～3.80）µmol·L^−1^·h^−1^，Ⅲ型患者GBA活性0.35（0.30～0.40）µmol·L^−1^·h^−1^。13例患者进行Lyso-GL1检测，92.3％（12/13）的患者Lyso-GL1水平明显增高（>386.7 ng/ml）。

3. GBA基因突变分析：20例患者共检出15种基因突变（表2），共计42个突变位点数，包括错义突变和无义突变，按照ACMG变异指南，S395F、L303I、K196R被认定为临床意义未明变异，其余均为致病变异。纯合突变6例、复合杂合突变14例。Ⅰ型和Ⅲ型患者L483P突变频率最高（35.7％，15/42），其次为V414L（16.7％，7/42）、L303I（9.5％，4/42）。4例Ⅲ型患者突变分别为L483P/F252I、L483P/D448H/N227S、L483P/L483P、N227S/N227K/V230G，均为热点突变。

**表2:** 20例戈谢病患者基因突变、酶活性以及生物标志物结果

例号	基因型	变异	氨基酸改变	葡萄糖脑苷脂酶活性（µmol·L^−1^·h^−1^）	葡糖鞘氨醇（ng/ml）
1	纯合	c.1240G>C	p.Val414Leu（V414L）	1.60	541.9
2	纯合	c.1240G>C	p.Val414Leu（V414L）	0.40	-
3	杂合	c.1448T>C c.754T>A	p.Leu483Pro（L483P） p.Phe252Ile（F252I）	0.30	-
4	杂合	c.1488T>C c.928A>G	p.Leu483Pro（L483P） p.Ser310Gly（S310G）	3.80	-
5	杂合	c.1448T>C c.1102C>T	p.Leu483Pro（L483P） p.Arg368Cys（R368C）	1.80	541.9
6	杂合	c.1448T>C c.1240G>C	p.Leu483Pro（L483P） p.Va1414Leu（V414L）	0.92	>400.0
7	杂合	c.1448T>C c.587A>G	p.Leu483Pro（L483P） p.Lys196Arg（K196R）	0.23	-
8	杂合	c.1448T>C c.1342G>C c.680A>G	p.Leu483Pro（L483P） p.Asp448His（D448H） p.Asn227Ser（N227S）	0.31	75.7
9	纯合	c.1488T>C	p.Leu483Pro（L483P）	0.40	386.7
10	杂合	c.1448T>C c.155C>T	p.Leu483Pro（L483P） p.Ser52Leu（S52L）	0.45	-
11	杂合	c.1448T>C c.907C>A	p.Leu483Pro（L483P） p.Leu303Ile（L303I）	0.59	>400.0
12	纯合	c.1240G>C	p.Val414Leu（V414L）	0.60	>400.0
13	杂合	c.907C>A c.703T>C	p.Leu303Ile（L303I） p.Ser235Pro（S235P）	0.38	>400.0
14	纯合	c.907C>A	p.Leu303Ile（L303I）	2.65	>400.0
15	杂合	c.1448T>C c.1342G>C	p.Leu483Pro（L483P） p.Asp448His（D448H）	0.43	-
16	杂合	c.1259G>A c.1184C>T	p.Trp420*（W420*） p.Ser395Phe（S395F）	0.33	-
17	杂合	c.680A>G c.681T>G c.689T>G	p.Asn227Ser（N227S） p.Asn227Lys（N227K） p.Val230Gly（V230G）	0.40	>400.0
18	纯合	c.1448T>C	p.Leu483Pro（L483P）	0.40	>400.0
19	杂合	c.1448T>C c.754T>A	p.Leu483Pro（L483P） p.Phe252Ile（F252I）	0.37	>400.0
20	杂合	c.1448T>C c.754T>A	p.Leu483Pro（L483P） p.Phe252Ile（F252I）	0.42	>400.0

注 -为未查或不详；基因定位NC_000001.11（155234452..155244627）

## 讨论

目前全球数据表明，GD Ⅰ型占95％、Ⅱ型占1％、Ⅲ型占2％～3％，而我国Ⅱ型、Ⅲ型比例高达30％～50％[2]，这主要与GBA基因突变型有关。

截至目前，人类基因突变数据库（HGMD）共收录GBA基因突变504种，包含错义突变、无义突变、剪接突变、缺失/插入突变、基因重排等，其中错义突变最常见（约占75.2％）。GBA基因突变具有种族差异，德系犹太人中N370S、c.84dupG、L483P和IVS2+l突变占95％，其中N370S占78％[3]。我国已发现GBA基因突变约40种，以L483P突变最常见（30.0％～47.7％），各型GD患者中均可见，其次为F252I、N227S、V414L突变[2],[4]。

本研究20例GD患者中，仍以L483P突变最为常见，占比35.7％，然后依次为V414L、L303I、F252I突变。研究表明，L483P突变可使GBA活性降低或缺失，纯合型与Ⅱ型、Ⅲ型GD相关[5]。本研究中，携带L483P突变Ⅰ型GD患者10例、Ⅲ型GD患者3例，其中2例L483P纯合型患者：1例为Ⅰ型GD，发病年龄早，但无神经系统受累症状；1例为Ⅲ型GD，合并双耳听力丧失、戈谢瘤（Gaucheroma）、蛋白丢失性肠病（Protein losing enteropathy，PLE）。PLE为GD罕见并发症，提示疾病预后差、死亡率高[6]。携带V414L突变4例，均为Ⅰ型GD患者，临床表现仅为脾大、血小板减少和（或）骨损害，临床症状轻，与既往报道一致[5]。L303I突变3例，也均为Ⅰ型GD患者，该突变于2018年首次在我国儿童GD中报道[7]，基因型与表型相关性尚未明确。此外，3例F252I/L483P突变患者，其中1例为Ⅲ型GD，神经症状表现为癫痫大发作，其余2例为儿童，目前尚未发现神经系统受累症状，同既往报道[4]F252I突变与严重的神经病变型之间相关存在差异。S310G突变为本课题组前期发现并首次报道的突变，通过蛋白结构分析，发现其对GBA活性影响较小[8]。K196R突变为中国人群首次报道，国外也仅1例GD合并胃癌患者的报道，其致病性尚未明确[9]。

根据临床表现分析，本研究中20例GD患者均有脾大（巨脾16例）和血小板减少，以轻中度血小板减少多见（16/20），且出血症状轻，机制可能与继发性脾功能亢进和骨髓浸润导致巨核细胞生成减少有关[10]。骨骼受累为GD常见表现，但轻重程度不一，严重者可造成不可逆损害，具有致残性[11]，脾切除后可加重骨骼系统受累[12]。本研究5例患者出现骨痛（1例V414L纯合突变、3例L483P杂合突变、1例N227S/N227K/V230G复合杂合突变），3例严重骨痛患者有脾切除史；16例患者X线或MRI提示骨损害，37.5％ GD患者影像学表现为烧瓶样改变，可见影像学改变早于骨痛症状出现，与既往文献报道一致[12]，提示GD要重视影像学检查，早期发现骨受累情况。此外，神经系统受累也是我国GD患者易见的临床表现。本研究中4例伴有神经病变型患者初诊均为Ⅰ型，在诊断后的1～18年确诊为Ⅲ型。目前，Ⅰ型GD和早期Ⅲ型GD临床表现重叠，且中枢神经系统受累早期症状不典型，易漏诊。GD患者诊断、治疗和随访中应及时评估神经病变，包括眼球运动、脑电图、听觉脑干诱发电位等，同时关注基因型与神经病变的关系。本研究中2例N227S复合杂合突变患者均在病程中出现癫痫大发作，提示N227S突变可能参与严重神经病变的发生[13]。

GD患者发生并发症的风险远高于健康人。有研究表明[14]，GD罹患恶性肿瘤风险增加1.4～9倍，多见于血液系统恶性肿瘤（多发性骨髓瘤、非霍奇金淋巴瘤），其次为肝癌、肾癌、黑色素瘤等实体瘤。本研究中1例L303I纯合型GD患者合并子宫颈癌，国际上也有1例相关报道[14]_。_此外，Ⅰ型GD与PD关系也尤为密切。目前已在PD患者发现130种GBA突变，为PD患者高风险遗传因素[15]；而携带GBA突变合并PD患者，发病年龄更早、临床症状更严重[16]。本研究也有1例GD（L483P/R368C复合杂合突变）患者合并PD，疾病早期即出现认知障碍，严重影响生活质量。

综上所述，需提高GD认识，对脾大、血小板减少高危患者进行早期筛查，实现早期诊断；L483P突变为中国GD患者常见突变，可能与神经病变相关，各基因型与临床表型之间仍具有不确定性。
